# Primary breast lymphoma mimicking triple-negative breast cancer: a case report with clinical and pathological implications

**DOI:** 10.1186/s40792-024-02032-3

**Published:** 2024-10-08

**Authors:** Toyoaki Sawano, Masahiro Wada, Akihiko Ozaki, Akinori Hashiguchi, Shinichi Hirooka, Tetsuya Tanimoto

**Affiliations:** 1 Department of Surgery, Jyoban Hospital of Tokiwa Foundation, 57 Kaminodai, Jyoban-Kami-Yunagayamachi, Iwaki, Fukushima Japan; 2 Breast and Thyroid Center, Jyoban Hospital of Tokiwa Foundation, Iwaki, Fukushima Japan; 3 Department of Breast Surgery, Utsunomiya Central Clinic, Utsunomiya, Tochigi Japan; 4 Department of Pathology, Keio University School of Medicine, Shinjuku, Tokyo, Japan; 5 Department of Pathology, Jyoban Hospital of Tokiwa Foundation, Iwaki, Fukushima Japan; 6 Department of Internal Medicine, Jyoban Hospital of Tokiwa Foundation, Iwaki, Fukushima Japan

**Keywords:** Lymphoma of the breast, Diffuse large B-cell lymphoma, Extranodal lymphoma, Carcinoma, Triple-negative breast cancer

## Abstract

**Background:**

Primary breast lymphoma (PBL) is a rare type of extranodal lymphoma, the diagnostic process for which presents significant challenges owing to an overlap in clinical and pathological features with those observed in triple-negative breast cancer (TNBC). However, the current literature reveals a paucity of information regarding the ramifications of potential diagnostic errors, particularly in the context of emergent therapeutic strategies for TNBC. Thus, we present a unique report of a case of PBL.

**Case presentation:**

A 76-year-old female with no past medical or family history presented to the hospital with the chief complaint of a mass in the right breast. Two masses were palpated in the right breast: one 56 mm mass (No. 1) located at 10 o'clock, and a 21 mm large, elastic, hard mass (No. 2) at 4 o'clock. Needle biopsy was performed only on the larger 56 mm mass (No. 1). The results showed invasive carcinoma that was negative for estrogen receptor, progesterone receptor, and human epidermal growth factor receptor-2. The preoperative diagnosis was right breast cancer (T3N0M0 Stage IIB) of the TNBC subtype. The patient refused the preoperative chemotherapy recommended by the treatment team; therefore, right breast mastectomy and sentinel lymph-node biopsy were performed instead. The histopathological diagnosis of the first mass was diffuse large B-cell lymphoma (DLBCL); that of the second mass (No. 2) was an invasive breast carcinoma of no special type. Postoperative treatment consisted of endocrine therapy (letrozole) for breast cancer, while the DLBCL was treated with chemotherapy and three courses of intrathecal chemotherapy. At the time of this report, the patient is still living, and neither tumor had recurred in the 2 years following surgery.

**Conclusions:**

On rare occasions, PBL can preoperatively mimic TNBC. While this case did not lead to serious consequences, because surgery was eventually selected as the first therapy, clinicians should be aware that the diagnosis of PBL is challenging using only a core-needle biopsy and can often be misdiagnosed as TNBC.

## Background

Triple-negative breast cancer (TNBC) is characterized by the negative expression of estrogen receptor (ER), progesterone receptor (PR), and human epidermal growth factor receptor-2 (HER2) [1, 2]. Because TNBC has clinical features of high invasiveness, high metastatic potential, propensity for relapse, and poor prognosis [2], ongoing efforts to improve treatment algorithms to improve patient outcomes are underway. Therefore, neoadjuvant chemotherapy with or without immune checkpoint inhibitors and response-reactive additional adjuvant chemotherapy has recently become the standard treatment strategy [3, 4]. However, insufficient information exists regarding whether there are any drawbacks to the latest treatment strategy for TNBC.

In this context, a case worth considering is primary breast lymphoma (PBL) [5–7], which is a rare form of extranodal lymphoma [5] with a frequency of only 0.13% within all malignant breast tumors [8]. PBL was first described by Wiseman and Liao (1972) [9] and is defined as a lymphomatous infiltration of the breast or nearby mammary tissue with extension only to the ipsilateral axillary lymph nodes [9]. PBL is a type of non-Hodgkin lymphoma, and its most common clinical feature is a painless breast mass without the B symptoms (fever, weight loss, and night sweats) that are typical of Hodgkin lymphoma. In the published literature, the 5-year overall survival rates for PBL patients with indolent and aggressive diseases were 75 and 54%, respectively [10]. Generally, treatment for PBL typically involves a 4–6 course of the R-CHOP (Rituximab, Cyclophosphamide, Doxorubicin, Vincristine, and Prednisone) regimen as chemoimmunotherapy, depending on the stage [11]. Moreover, standard histopathological techniques such as hematoxylin and eosin (H&E) staining along with routine immunostaining (ER, PR, and HER2) are inadequate for diagnosing PBL, as this cancer type requires additional immunostaining markers such as CD20 for accurate identification.

Consequently, diagnosis of PBL reportedly presents significant challenges owing to an overlap in clinical and pathological features with those observed in breast cancer, and notably TNBC [12, 13]. However, the existing literature reveals a paucity of information regarding the ramifications of potential diagnostic errors, particularly in the context of the latest therapeutic strategies for TNBC. Here, we describe our encounter with a patient with PBL that mimicked TNBC. The preoperative diagnosis was TNBC, but the surgical diagnosis was PBL. It is crucial to perform a thorough analysis of cases such as this, coupled with an in-depth examination of the diagnostic complexities inherent in PBL, along with the potential consequences of diagnostic errors. Such an analysis is essential for the prompt and precise diagnosis and treatment of both PBL and TNBC within contemporary clinical frameworks.

## Case presentation

**Fig. 1:**
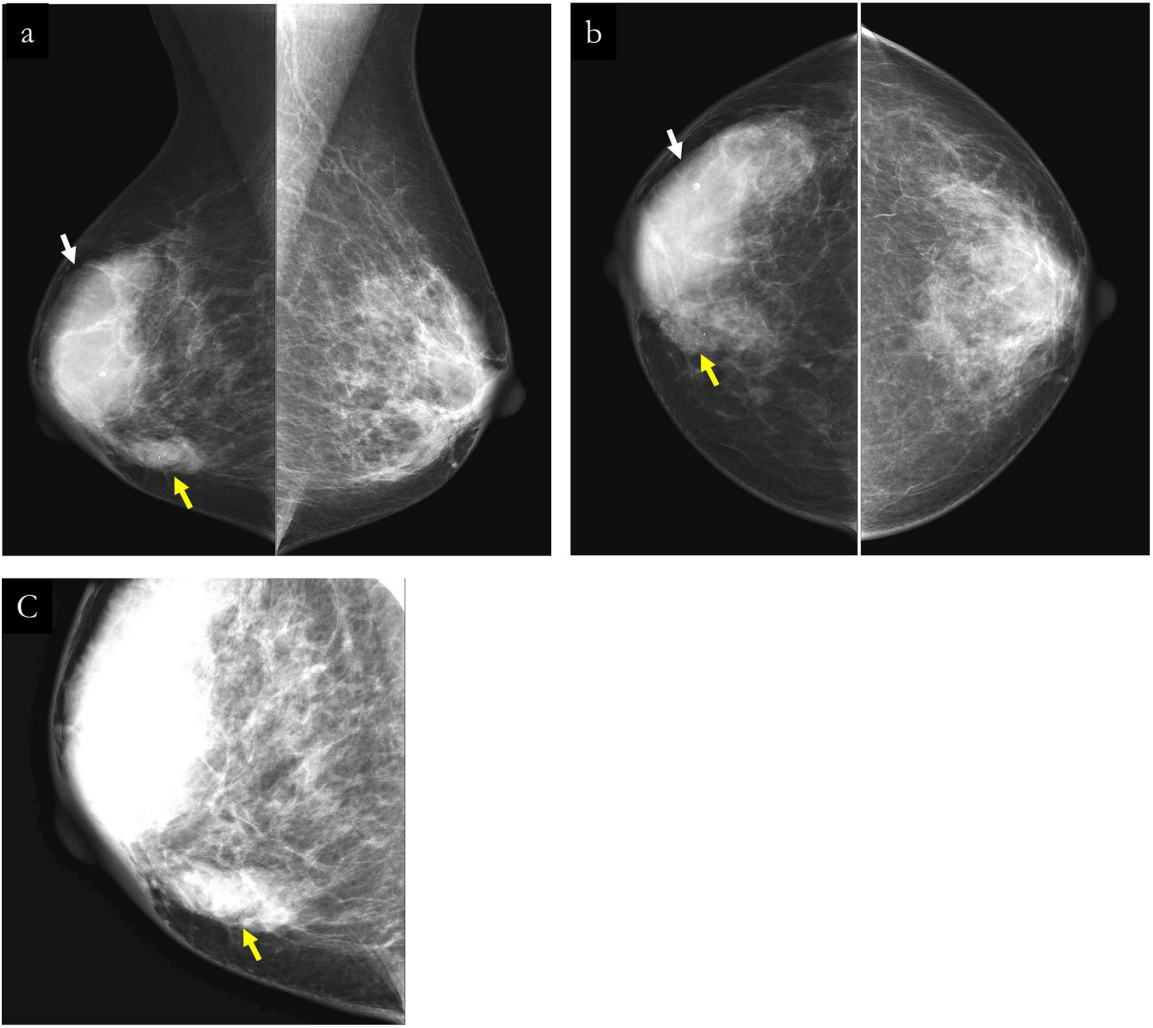
Mammography. **a**, **b** displays the mediolateral oblique and craniocaudal views of the right breast mammography, respectively. Mass No. 1, indicated by a white arrow, is characterized as an oval, well-circumscribed, smooth-contoured, high-density lesion. In contrast, mass No. 2, marked by a yellow arrow, is depicted as an oval mass with an indistinct margin and high density. **c** displays an enlarged mediolateral oblique view

**Fig. 2:**
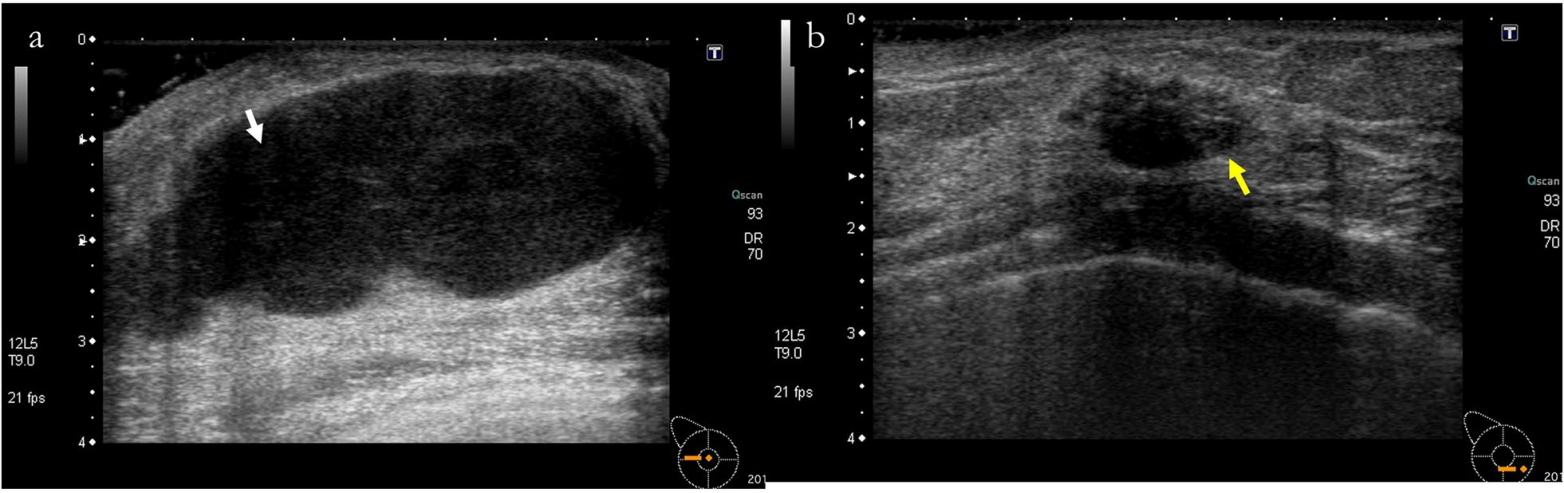
Ultrasonography. **a** illustrates mass No. 1, characterized as a lobulated, well-defined, smooth-edged, hypoechoic lesion with a size of 56 mm located on the lateral aspect of the right breast. **b** depicts mass No. 2, which presents as an irregular, well-defined, and rough-edged hypoechoic lesion measuring 28 mm and situated on the medial aspect of the right breast

**Fig. 3:**
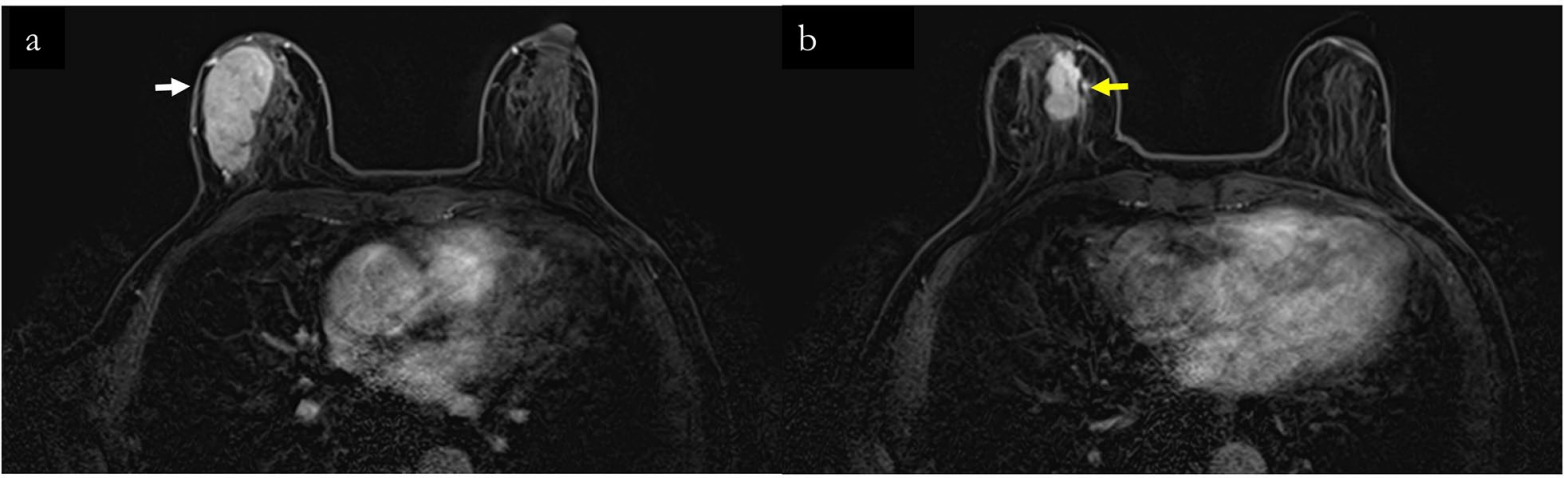
Contrast-enhanced breast magnetic resonance imaging findings. **a** Mass No. 1 is depicted as a lobulated lesion with a somewhat irregular but distinct boundary, measuring 53 mm and located at the 9 o’clock position of the right breast. The lesion demonstrates a fast washout pattern on the time–intensity curve. Conversely, **b** illustrates mass No. 2 with a diameter of 26 mm situated in the inferomedial quadrant of the same breast. This mass is characterized by a border that is less well defined compared to mass No. 1 and exhibits a fast washout pattern on the time-intensity curve

**Fig. 4:**
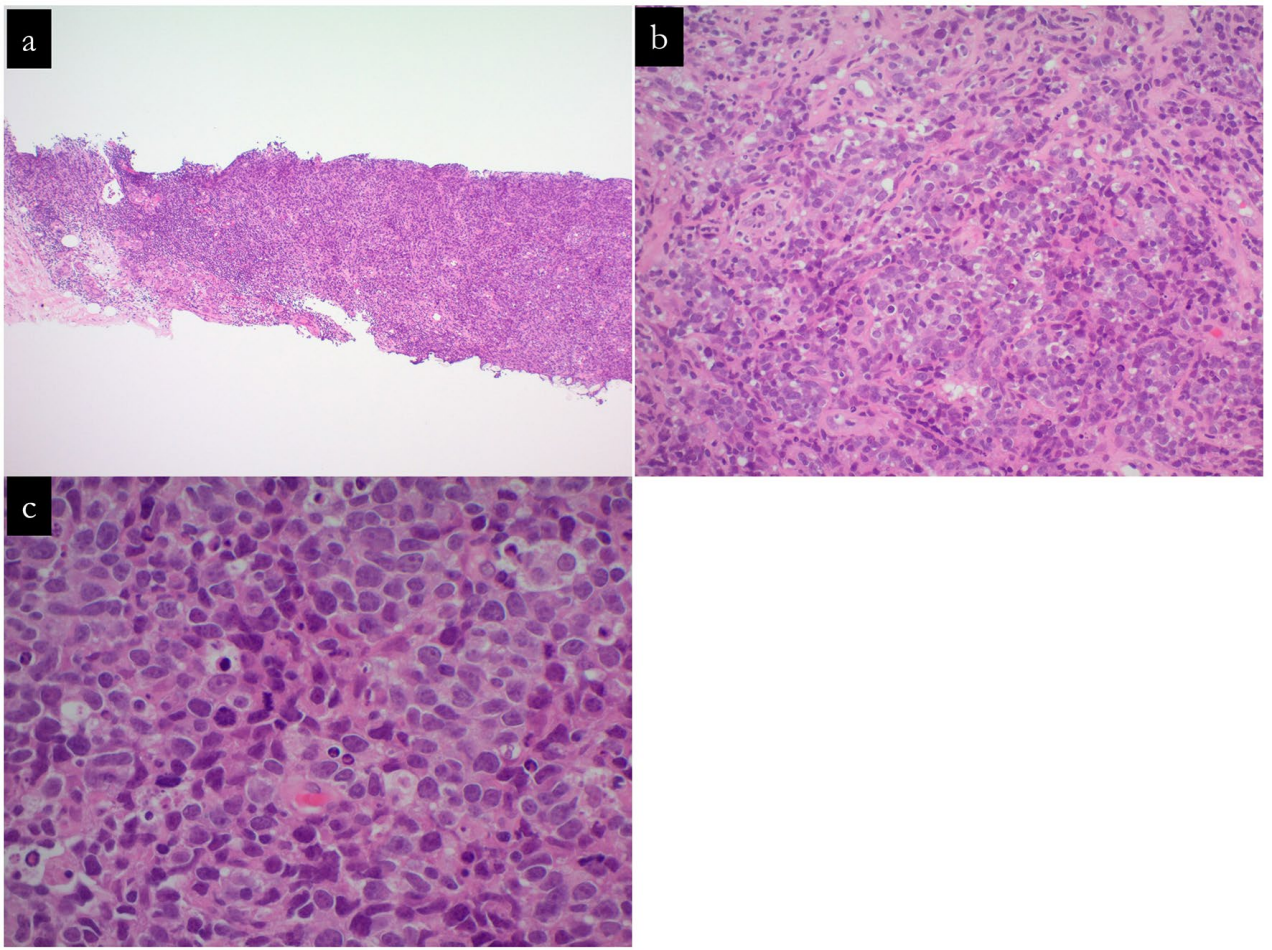
Hematoxylin and eosin-stained sections from core-needle biopsy of specimen No. 1. **a–c** depicts the neoplastic tissue at ×5, ×200, and ×400 magnifications, respectively. The diagnosis of mass No. 1 as invasive carcinoma is substantiated by the histopathological features of a poorly differentiated neoplasm exhibiting solid growth patterns with occasional alveolar structures. The tumor is assigned a nuclear grade of 3 and a histological grade of 3. Immunohistochemical staining results are negative for estrogen receptor and progesterone receptor, with a human epidermal growth factor 2 score of 0. The Ki-67 proliferation index is markedly elevated at 100%

A 76-year-old female with no past medical or family history presented to the hospital with the chief complaint of a right breast mass. During physical examination, two masses were palpated in the right breast. The first (No. 1) was a 56 mm right breast mass at the 10 o'clock position, while the second (No. 2) was a 21 mm large elastic hard mass at the 4 o’clock position. Imaging studies suggested the masses to be breast cancer as both showed compressible growth (Figs. 1, 2, 3). Core needle biopsy was performed on only the 5-cm-sized mass (No. 1) (Fig. 4). The results indicated an invasive breast carcinoma of no special type (NST) that was negative for ER, PR, and HER2. It was a poorly differentiated carcinoma with focal growth. Additionally, the other mass (No. 2) was clinically diagnosed as the same tumor as the first mass, but a needle biopsy was not performed.

The preoperative diagnosis was right breast cancer (T3N0M0 Stage IIB) of the TNBC subtype. The treatment team recommended preoperative chemotherapy, but the patient refused; therefore, surgical treatment (right breast mastectomy and sentinel lymph-node biopsy) was performed initially. The surgical and postoperative courses were uneventful, and the patient was discharged on postoperative day 8.

**Fig. 5:**
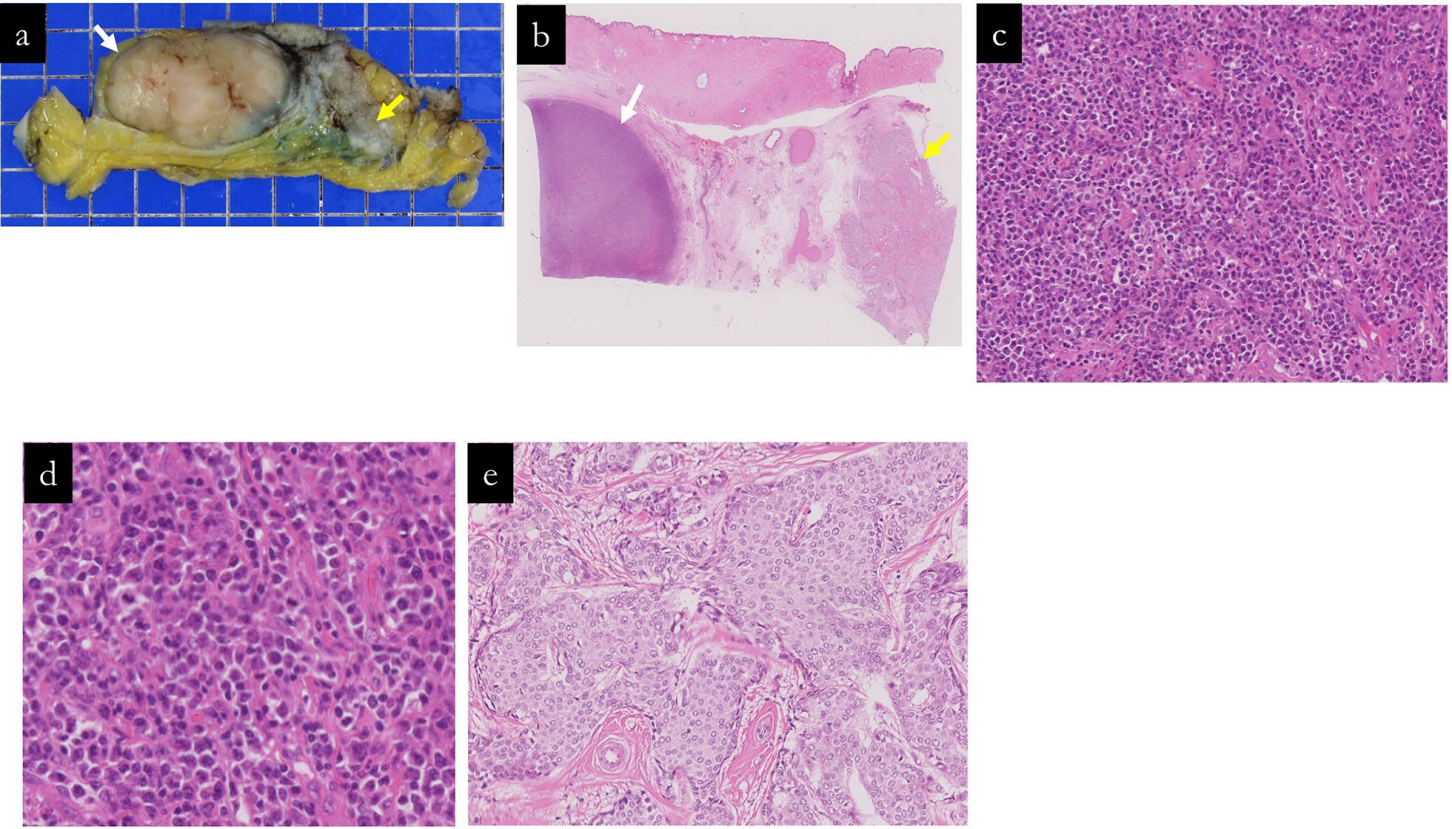
Pathological findings of surgical specimens No. 1 and No. 2. Fresh and formalin-fixed sections are shown in (**a**). Specimen No. 1 is described as a milky white, nodular mass, whereas specimen No. 2 is a milky white, lobulated mass; both are discrete without any interconnection. **b–e** displays escalating magnifications of Hematoxylin and Eosin staining, highlighting the homogeneous cell proliferation with a pronounced nucleus-to-cytoplasm ratio in specimen No. 1. Specimen No. 2 is typified by the classic hallmarks of invasive breast carcinoma of no special type featuring a solid growth pattern and nested infiltration

**Fig. 6:**
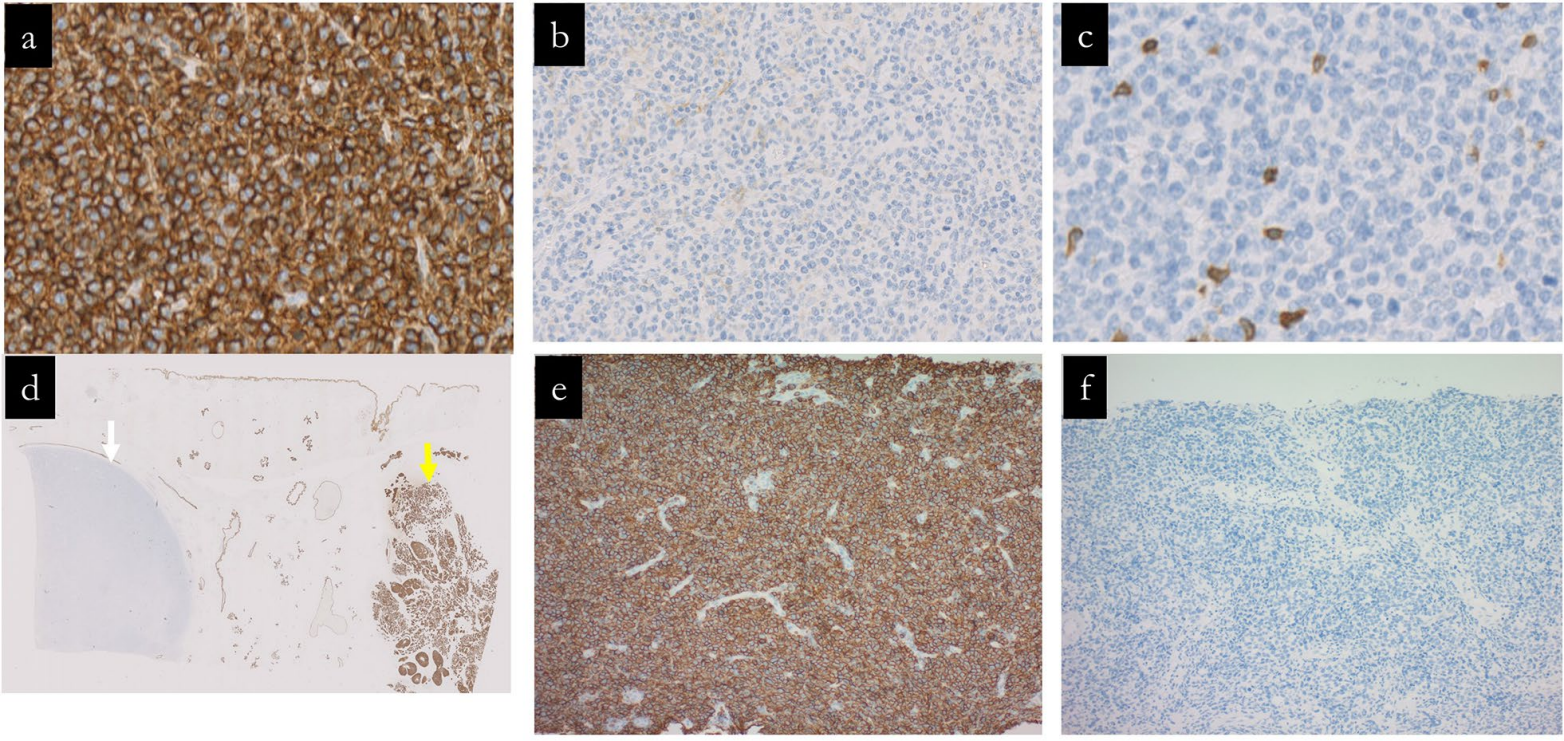
Immunohistochemical profiling of surgical specimens No. 1 and No. 2 and core-needle specimens. **a–c** sequentially exhibit the immunoreactivity of mass No. 1 to CD20, CD10, and CD3 markers, each at ×200 magnification. **d** compares masses No. 1 and No. 2, demonstrating their staining patterns with cytokeratin AE1/AE3 at ×5 magnification. **e**, **f** exhibits the immunoreactivity of the core-needle biopsy specimen to CD20 and cytokeratin AE1/AE3 markers, respectively, each at ×100 magnification

The histopathology results of the surgical specimen showed a malignant lymphoma with a tumor size of 38 × 17 mm, CD20 (+), CD45 (+), CD79a (+), CD3 (−), CD10 (−), and pancytokeratin (−), indicating diffuse large B-cell lymphoma (DLBCL). In contrast, the other tumor (No. 2) was an invasive breast carcinoma of NST, ER (+), PR (+), HER2 score: 0, pT2 (21 mm), and pN0 (sentinel lymph node). Pathologically, no continuity was found between the two tumors (Figs. 5, 6). We reassessed the core-needle biopsy specimen, noting that the tumor cells displayed a more lymphoid morphology than previously observed, which may be attributable to variabilities in tissue preparation, including potential artifacts introduced during needle biopsy and tissue fixation. Due to the diffuse proliferation of large lymphoid cells, immunostaining was conducted under the suspicion of malignant lymphoma. The tumor cells tested positive for CD20, CD45, CD79α, BCL2, and BCL6, and negative for CD3, CD5, CD10, cyclin D1, and cytokeratin AE1/AE3. These findings confirmed the diagnosis of diffuse large B-cell lymphoma (DLBCL) (Fig. 6).

Postoperative therapy consisted of endocrine therapy (letrozole) for breast cancer. For DLBCL, the patient was determined to be in Stage 1E after a systemic search and was treated with five courses of R-CHOP chemotherapy followed by three courses of intrathecal chemotherapy with methotrexate, cytarabine, and dexamethasone. At 2 years post-surgery, the patient was alive, and neither tumor had recurred.

## Discussion

This case represents a rare occurrence in which a preoperative needle biopsy revealed TNBC, but the final diagnosis of the surgical specimen simultaneously revealed PBL and a separate breast cancer in the same breast. In addition to the inherent diagnostic challenges presented by core-needle biopsy, this case underscores the potential clinical repercussions stemming from such diagnostic inaccuracies, namely an initiation of incorrect chemotherapy. To prevent such misdiagnoses and unwanted consequences, clinicians and pathologists should consider the possibility of PBL in the preoperative diagnosis of TNBC if certain conditions are met.

Our experience shows that PBL can be incorrectly diagnosed as TNBC by needle biopsy, and incorrect treatment may be initiated. The diagnosis of a breast mass is typically made using multifocal imaging studies and histopathological diagnosis by needle biopsy. However, typical clinical and pathological features are insufficient to differentiate PBL from TNBC [14]. In this case, although two non-contiguous masses are present in the unilateral breast, it is considered that these masses are related, namely daughter nodes, as clinicians frequently encounter such situations. From the pathologist's perspective, the biopsy specimen revealed a poorly differentiated tumor; however, the tumor cells exhibited epithelioid connections and displayed alveolar growth with minimal stroma. Given that primary malignant lymphoma of the breast is rare and not clinically suspected, this diagnosis was not considered. Therefore, clinicians should consider the possibility of PBL that presents as poorly differentiated carcinoma when a patient's neoplasm is poorly differentiated on H&E staining and exhibits negative results for both ER/PR and HER2 on immunostaining. Conversely, when the tumor is poorly differentiated, "triple negative," and exhibits a high Ki-67 index, the pathologist should consider malignant lymphoma in the differential diagnosis. In such cases, further immunostaining, including CD20, is advised for a definitive diagnosis. Furthermore, in addition to immunostaining after needle biopsy to make an earlier diagnosis, deciding on a surgery-first strategy may be useful when PBL is suspected. Moreover, in this specific case, needle biopsies of both lesions, despite their location within the same mammary gland, may have been useful for obtaining a more accurate preoperative diagnosis as they were not contiguous.

In this case, the diagnosis of PBL was not made preoperatively; however, the introduction of chemotherapy was delayed owing to the patient's preference for surgery-first treatment. Conversely, if neoadjuvant chemotherapy had been administered, the regimen of neoadjuvant chemotherapy for TNBC would have likely been dose-dense adriamycin–cyclophosphamide (AC) combination chemotherapy. Because AC combination chemotherapy overlaps with some of the drugs in R-CHOP chemotherapy, it may have resulted in a partial response, making interpretation more difficult and indicating the importance of suspecting PBL instead of TNBC.

## Conclusions

This report presents a rare case of PBL that preoperatively mimicked TNBC. Although the difficulty in diagnosis did not lead to serious consequences, because surgery was eventually selected as the first therapy, clinicians should be aware that the diagnosis of PBL is challenging using only a core-needle biopsy and can potentially be misdiagnosed as TNBC.

## Data Availability

Patient information is recorded in the hospital medical records, and the data are considered confidential by the Privacy Act.

## Abbreviations

AC: Adriamycin–cyclophosphamide
DLBCL: Diffuse large B-cell lymphoma
ER: Estrogen receptor
HE: Hematoxylin and eosin
HER2: Human epidermal growth factor receptor-2
PBL: Primary breast lymphoma
PR: Progesterone receptor
TNBC: Triple-negative breast cancer
